# GESim: ultrafast graph-based molecular similarity calculation via von Neumann graph entropy

**DOI:** 10.1186/s13321-025-01003-6

**Published:** 2025-04-22

**Authors:** Hiroaki Shiokawa, Shoichi Ishida, Kei Terayama

**Affiliations:** 1 Center for Computational Sciences, University of Tsukuba, Tennodai 1-1-1, Tsukuba, Ibaraki 305-8577 Japan; 2 MolNavi LLC, #402 Wizard building 1-4-3 Sengen-cho Nishi-ku, Yokohama, Kanagawa 220-0072 Japan; 3 Graduate School of Medical Life Science, Yokohama City University, 1-7-29 Suehiro-cho, Tsurumi-ku, Yokohama, Kanagawa 230-0045 Japan

## Abstract

Representing molecules as graphs is a natural approach for capturing their structural information, with atoms depicted as nodes and bonds as edges. Although graph-based similarity calculation approaches, such as the graph edit distance, have been proposed for calculating molecular similarity, these approaches are nondeterministic polynomial (NP)-hard and thus computationally infeasible for routine use, unlike fingerprint-based methods. To address this limitation, we developed GESim, an ultrafast graph-based method for calculating molecular similarity on the basis of von Neumann graph entropy. GESim enables molecular similarity calculations by considering entire molecular graphs, and evaluations using two benchmarks for molecular similarity suggest that GESim has the ability to differentiate between highly similar molecules, even in cases where other methods fail to effectively distinguish their similarity. GESim is provided as an open-source package on GitHub at https://github.com/LazyShion/GESim.

## Scientific Contribution

We developed GESim, an ultrafast graph-based method for calculating molecular similarity on the basis of von Neumann graph entropy. We extended von Neumann graph entropy, a traditional graph-based measure of structural complexity, to perform efficient molecular similarity calculations without sacrificing its strong capability to distinguish structurally different molecules. While graph-based similarity calculation approaches are typically computationally demanding, GESim enables similarity calculations to be performed at a cost comparable to that of fingerprint-based approaches.

## Introduction

Molecular similarity is a fundamental concept in chemoinformatics and medicinal chemistry and is widely used in applications ranging from database searches to virtual screening[1–5]. In database searches, molecular similarity often refers to structural similarity, retrieving structurally similar molecules, whereas in virtual screening, it often focuses on functional similarity, selecting structurally different molecules with similar biological activity. To quantify molecular similarity, molecular fingerprints are used as standard molecular representations, encoding structural features either as bits in a bit string or as counts in a vector[6, 7], and are employed in conjunction with similarity and distance metrics, such as the Tanimoto index and cosine coefficient[8]. Two-dimensional (2D) fingerprints are commonly used for molecular similarity calculations because of their efficiency and simplicity[9–12]. The term “2D” in 2D fingerprints means that these fingerprints encode molecular structural features based on topological information, which describes the connectivity of atoms and bonds, without considering spatial information, such as atomic coordinates or molecular conformations. 2D fingerprints can be categorized as dictionary-based, topological- or path-based, circular-based, or pharmacophore-based fingerprints; notable examples include molecular access system keys (MACCS)[9], atom-pair fingerprints (APFP)[10], topological-torsion fingerprints (TTFP)[11], extended-connectivity fingerprints (ECFP)[12], and feature-connectivity fingerprints (FCFP)[12], respectively. String representations, molecular graph representations, and three-dimensional (3D) molecular representations have also been utilized as other types of molecular representations[6, 13–15]. Although certain combinations of molecular representations and similarity metrics, such as ECFP combined with the Tanimoto index, perform better in various tasks related to molecular similarity, each combination excels in certain tasks and underperforms in others, indicating that no single combination is universally optimal[16, 17].

Methods for directly computing molecular similarity from molecular graphs, rather than through fingerprints or descriptors, have gained increasing attention in recent years[18–20], along with the advancements and promising performances of deep learning techniques utilizing graph representations[21–27]. The approach of treating a molecule as a graph, where atoms are nodes and bonds are edges, captures the intricate topological and overall structural features of molecules that are not fully considered by conventional methods, such as fingerprint-based methods. In chemoinformatics and medical chemistry, the graph edit distance (GED) has been proposed as a graph-based method for calculating molecular similarity. For example, GED-based similarity search has demonstrated promising performance in virtual screening tasks [18]. Although GED is effective for evaluating molecular similarity, the calculation of GED is computationally demanding because it is performed in $\mathcal {O}(n^3)$ time, where *n* is the number of atoms in a molecule. To address this computational challenge, filter-and-verification approaches have been developed for GED in recent years [28–32]. However, as reported by Naoi et al. [33], the search efficiency of this approach is much less than that of fingerprint-based methods. More specifically, it has been reported that GED would be feasible for small molecules with around 16 heavy atoms. Thus, there is a strong need to find an effective graph-based similarity method that achieves efficient and accurate search performance simultaneously.

Given this situation, we propose GESim, an ultrafast graph-based method for calculating molecular similarity that is based on von Neumann graph entropy (vNGE). vNGE quantifies structural complexity of a graph by extracting spectral features from it, which represent connectivity among nodes [34]. Owing to its effectiveness, vNGE has recently been used in many applications in graph structure analysis and pattern recognition, such as anomaly detection [35], link analysis [36], and others [37, 38]. Although the exact computation of vNGE is computationally expensive, GESim achieves high efficiency by employing the one-dimensional structural information [39], defined as the Shannon entropy of the normalized degree sequence, which provides a good approximation of vNGE within a short computation time [40]. Thus, GESim enables graph-based molecular similarity calculations at a computational speed comparable to that of fingerprint-based methods, overcoming the impractically high computational cost that hinders the use of graph-based methods in applications such as database searches and virtual screening tasks. Additionally, by using vNGE, GESim enables molecular similarity calculations to be performed by considering entire molecular graphs. To evaluate the characteristics of GESim, a structural similarity benchmark[17] and a functional similarity benchmark were used[16, 41], and the results suggested that GESim has the ability to differentiate between highly similar molecules, even in cases where other methods fail to effectively distinguish their similarity. Additionally, GESim provides a visualization function for atom-pair matching in a molecule pair, improving user understanding of the GESim calculation results. The open-source GESim package is available on GitHub at https://github.com/LazyShion/GESim.

## Methods

### Overview of GESim

GESim measures the graph-based similarity between two molecules via vNGE [34]. vNGE is a traditional graph-based measure that quantifies the structural complexity of a graph by extracting the spectral features of the graph. Since the spectral features effectively represent connectivity among nodes [42], vNGE is a promising tool for understanding how a graph is structured. In summary, vNGE effectively distinguishes two graphs that are similar but somewhat different in structure.

**Fig. 1:**
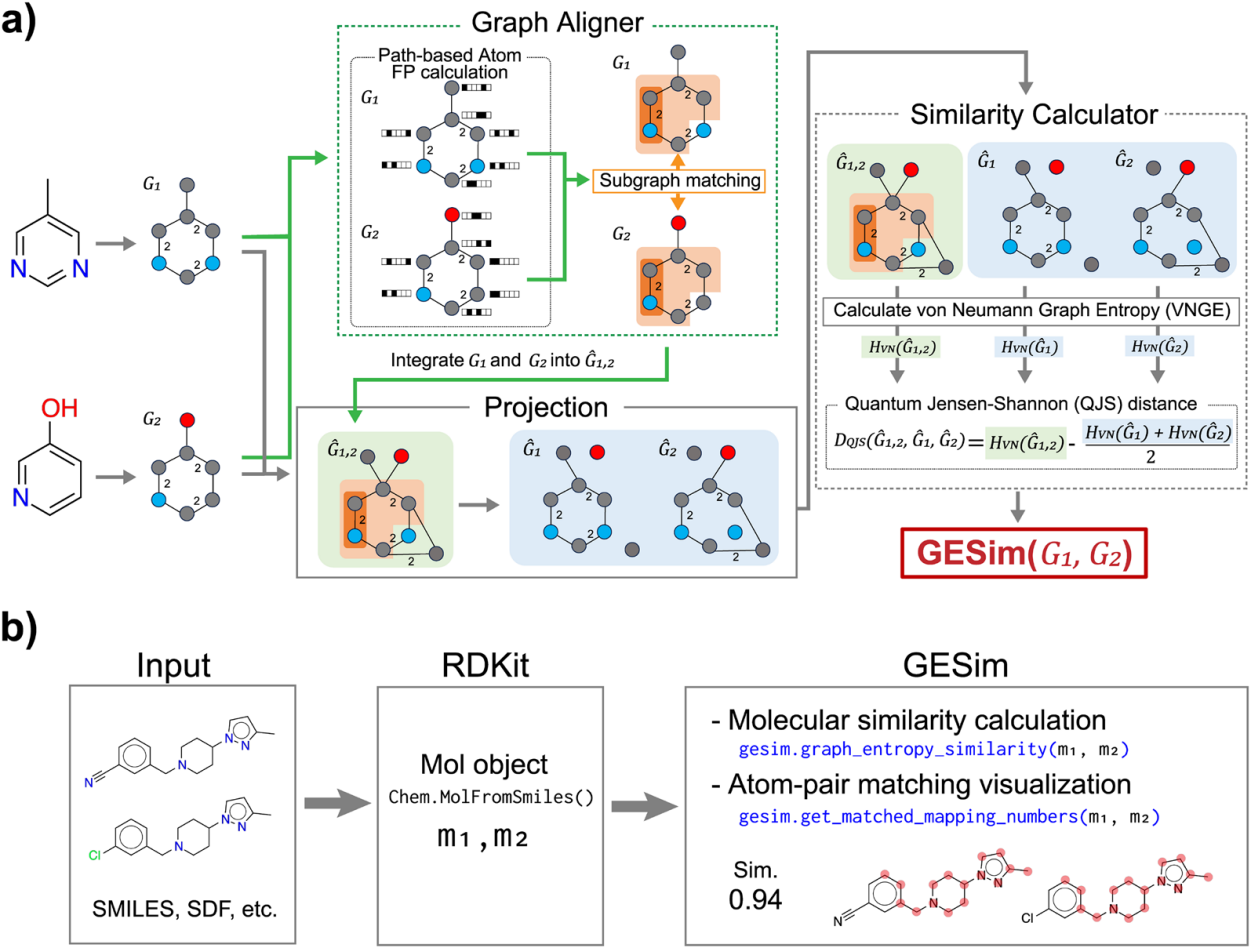
Overview of GESim.** a** GESim consists of three modules: Graph Aligner, Projection, and Similarity Calculator. The Graph Aligner module performs subgraph matching between two molecules: $G_1$ and $G_2$; the Projection module builds the merged graph $\hat{G}_{1, 2}$ and obtains $\hat{G_1}$ and $\hat{G_2}$ by projecting $G_1$ and $G_2$ onto $\hat{G}_{1, 2}$; and the Similarity Calculator module calculates the QJS distance via $\hat{G_1}$, $\hat{G_2}$, and $\hat{G}_{1, 2}$. **b** The figure shows the workflow of the Python program for calculating molecular similarity and visualizing subgraph matching via GESim

GESim extends vNGE to measure the structural differences between two molecules. Figure 1 (a) illustrates the whole process of GESim. As shown in the figure, GESim starts its calculation by converting input molecules into labeled graphs, $G_1$ and $G_2$. Then, GESim quantifies their similarity on the basis of vNGE by using Quantum Jensen-Shannon (QJS) divergence [43], which is a method of measuring the similarity between two entropies (*i.e.,* vNGEs of graphs.) QJS divergence requires three graphs to compute the similarity between $G_1$ and $G_2$ [40]. One is a merged graph $\hat{G}_{1, 2}$, which integrates $G_1$ and $G_2$. The other two are the graphs $\hat{G}_1$ and $\hat{G}_2$, which are projections of $G_1$ and $G_2$ onto $\hat{G}_{1, 2}$. To facilitate the computation of QJS divergence, GESim employs the following three steps, as shown in Fig. 1 (a): First, in the Graph Aligner module, GESim explores the largest common subgraph between $G_1$ and $G_2$ by computing atom-level matches on the basis of fingerprints and subgraph matches. In the Projection module, GESim generates $\hat{G}_{1, 2}$ by merging $G_1$ and $G_2$ on the basis of the common subgraph and projects $G_1$ and $G_2$ onto $\hat{G}_{1, 2}$ to construct $\hat{G}_1$ and $\hat{G}_2$, respectively. Next, in the Similarity Calculator module, GESim calculates the vNGEs of $\hat{G}_{1, 2}$, $\hat{G}_1$, and $\hat{G}_2$ and finally compares them via QJS divergence to quantify the similarity between $G_1$ and $G_2$. In the next subsection, we present detailed definitions of vNGE and QJS divergence, followed by a concrete description of each step.

As previously noted, we have published the open-source GESim package on GitHub, which provides RDKit-compatible Python functions, including similarity calculations and visualizations. Figure 1 (b) illustrates a specific use case of our package. Given molecules in a standard format such as SMILES or SDF, GESim receives Mol objects converted from the molecules via RDKit. For these inputs, $m_1$ and $m_2$, GESim provides the following two basic functions: The first is gesim.graph_entropy_similarity($m_1$, $m_2$), which evaluates the similarity between molecules $m_1$ and $m_2$ on the basis of vNGE. This function returns a similarity value ranging between 0 and 1, with values closer to 1 indicating that $m_1$ and $m_2$ are structurally similar. The second is gesim.get_matched_mapping_numbers($m_1$, $m_2$), which indicates the atom-pair matching between $m_1$ and $m_2$ extracted by the Graph Aligner module. As shown in Fig. 1 (a), the Graph Aligner module explores the largest common subgraph between $m_1$ and $m_2$ to facilitate QJS divergence. This function enables users to see the internal behavior of GESim during a similarity calculation, which can help them better understand the results. More specifically, this function reveals how GESim regards the two molecules as structurally similar.

### Molecular similarity calculation in GESim

GESim calculates the structural similarity between two molecules via QJS divergence, which compares the vNGEs of the molecules. In this section, we first introduce the basic notation and definitions used in GESim, followed by a step-by-step description of the similarity calculation process of GESim.

#### Basic notation and definitions

A molecule is modeled as a labeled graph $G=(V, E, \ell )$, where a node set *V* and an edge set *E* correspond to atoms and chemical bonds, respectively. $\ell$ is a label function that maps nodes and edges to corresponding chemical elements and bond types, respectively. In this study, $\ell$ is based on an atom code function used in the atom-pair fingerprints (APFP)[10], and bond types are obtained via RDKit[44]. For simplicity, we omit this label function $\ell$, and we denote $n = |V|$ and $m = |E|$ if their meanings are clear from the context. $A \in \mathbb {R}^{n \times n}$ represents the adjacency matrix of *G*, where $A_{ij} = 1$ if an edge $(v_i, v_j) \in E$; otherwise, $A_{ij} = 0$. We define the degree of $v_i \in V$ in *G* as $d_i = \sum _{j=1}^{n}A_{ij}$. Additionally, we introduce the Laplacian matrix of *G* as $L = D - A$, where *D* is a diagonal matrix such that $D = \text {diag}(d_1, d_2, \dots , d_n)$.

vNGE [34] is a spectral-based entropy measure that distinguishes the complexity of the structures of different graphs. For a given graph *G* and its Laplacian matrix *L*, the vNGE of *G* is the Shannon entropy of the rescaled spectrum derived from *L*. Formally, vNGE is given by the following definition:

##### Definition 1

(von Neumann Graph Entropy (vNGE)) Given a graph $G=(V, E)$ and its Laplacian matrix *L*, the vNGE of *G*, denoted as $\mathcal {H}_{\text {vn}}(G)$, is defined as

1 $$\begin{aligned} \mathcal {H}_{\text {vn}}(G) = {\left\{ \begin{array}{ll} - \sum _{i=1}^{n} \frac{\lambda _{i}}{\textit{vol}(G)} \log _{2}\left( \frac{\lambda _{i}}{\textit{vol}(G)} \right) & (\textit{vol}(G) > 0), \\ 0 & (\text {otherwise}),\\ \end{array}\right. } \end{aligned}$$

where $\lambda _{1} \ge \lambda _{2} \ge \dots , \ge \lambda _{n} = 0$ are the eigenvalues of *L* and $\textit{vol}(G) = \sum _{i=1}^{n}\lambda _i$.

On the basis of the spectra of the Laplacian matrix, vNGE effectively distinguishes different graph structures since the spectra are well-known to contain rich information about the inherent structural complexity of graphs, such as the connectivity and degree distribution of nodes. For example, vNGE is maximal if *G* is a complete graph, whereas it is minimal for *G* composed of only a single edge. If *G* forms a ring graph, vNGE yields intermediate scores between a complete graph and a single edge.

However, despite the strong capability to measure the structural complexity of graphs, vNGE has high computational costs, since computing the Laplacian spectra incurs $\mathcal {O}(n^3)$ time. To reduce this computational overhead, GESim employs one-dimensional structural information (SI) [39], denoted by $\mathcal {H}_{1}(G)$ for a graph *G*, instead of using Definition 1 directly. As reported by Liu et al. [40], SI effectively approximates vNGE by replacing the spectra of *L* in Definition 1 with the degrees of nodes. Specifically, SI is defined as follows:

##### Definition 2

(One-dimensional structural information (SI)) Given a graph *G*, the SI of *G*, denoted as $\mathcal {H}_{1}(G)$, is defined as

2 $$\begin{aligned} \mathcal {H}_{1}(G) = {\left\{ \begin{array}{ll} - \sum _{i=1}^{n} \frac{d_{i}}{\textit{vol}(G)} \log _{2}\left( \frac{d_{i}}{\textit{vol}(G)} \right) & (\textit{vol}(G) > 0), \\ 0 & (\text {otherwise}),\\ \end{array}\right. } \end{aligned}$$

where $$\textit{vol}(G) = \sum _{i=1}^{n}d_i$$.

Since the Laplacian spectra and degree are closely related in a graph *G*, the approximation error between SI and vNGE is tightly bounded in any unweighted graph [40]. Unlike the spectrum of the Laplacian matrix in Definition 1, the degree can be obtained in $\mathcal {O}(1)$ time. Specifically, Liu et al. reported that SI can compute the entropy at least two orders of magnitude faster than vNGE implemented using BLAS or LAPACK, even though it has almost no approximation error [40]. Hence, SI efficiently quantifies the structural complexity of a graph without sacrificing the strong graph discrimination capability of vNGE.

#### Similarity calculation process in GESim

Here, we present the similarity calculation process shown in Fig. 1 (a). On the basis of Definition 2, GESim measures the similarity between two molecules. As previously noted, GESim takes two graph-represented molecules, $G_1$ and $G_2$, as inputs; GESim then calculates their similarity via QJS divergence [43], which is a method of measuring the similarity between two entropies. In more detail, GESim calculates the similarity in the following three steps.

**(Step 1) Finding the largest subgraph matching:** To determine the QJS divergence between $G_1$ and $G_2$, in the Graph Aligner module, GESim extracts the largest subgraph matching between the two graphs: the nodes that are common between them. Traditionally, the maximum common structure (MCS) [45] approach is a natural choice for this purpose. However, this approach cannot be used to compute the similarity efficiently because MCS has intractable computational complexity. For this reason, GESim uses an approximate approach based on an atom fingerprint to extract the subgraph matching between two graphs. Specifically, GESim outputs the subgraph matching between two graphs by extracting all possible matching nodes as follows:

First, GESim calculates an individual atom fingerprint for every node in $G_1$ and $G_2$. Given a specific node *v* and a user-specified parameter *r*, the atom fingerprint $f_v$ is defined as a 1024-bit vector in which a set of unique edge paths rooted at the node has been hashed. To elaborate, GESim first enumerates unique paths of length 0 (node label) to *r* rooted at the node in the graph. Subsequently, GESim clusters these paths into sets of paths of identical length and hashes each of them into a bit. As a result, $r+1$ bits are placed throughout $f_v$. Unless otherwise stated, GESim employs the above atom fingerprint with $r=4$ as a default setting, but other types of atom fingerprints can be applied to GESim. For convenience, we denote the bit count of the result of a logical AND operation between two atom fingerprints, $f_{v_i}$ and $f_{v_j}$, as $|f_{v_i} \cap f_{v_j}|$.

Next, GESim extracts all node matches between $G_1$ and $G_2$ via the atom fingerprint according to the definition below.

##### Definition 3

(Node matching) Let $u \in \mathbb {N}$ be a user-specified parameter, and let $\overline{V}_2$ be a subset of nodes in $V_2$ that have not been matched with any node in $V_1$. Given two graphs $G_{1}(V_1, E_1)$ and $G_{2}(V_2, E_2)$, $v_i \in V_1$ is a match with $v_j \in V_2$ if and only if $v_j = \text {arg\ max}_{v \in \Theta (v_i, \overline{V}_2)} |f_{v_i} \cap f_{v}|$, where $\Theta (v_i, \overline{V}_2) = \{ v \in \overline{V}_2 \mid |f_{v_i} \cap f_{v}| \ge r- u\}$. If $v_i$ matches $v_j$, this node match is denoted as $v_i \leftrightarrow v_j$.

Definition 3 indicates that node $v_i$ in $G_1$ matches node $v_j$ in $G_2$ if $f_{v_i}$ and $f_{v_j}$ satisfy the following two conditions: (1) $|f_{v_i} \cap f_{v_j}|$ is greater than or equal to $r-u$, and (2) $f_{v_i}$ and $f_{v_j}$ have the largest $|f_{v_i} \cap f_{v_j}|$ in $\overline{V}_2$. Note that the node matching is symmetric; that is, if $v_i \leftrightarrow v_j$, then $v_j \leftrightarrow v_i$ holds as well. As shown in Fig. 1 (b), this node matching result can be visualized via a GESim function, $\texttt {gesim.get\_matched\_mapping\_numbers}(m_1, m_2)$.

**(Step 2) Generating merged and projected graphs:** In this step, GESim performs the projection to generate three special graphs on the basis of the subgraph matching obtained in Step 1. As mentioned above, QJS divergence requires three input graphs to compare the vNGEs of the two given graphs, $G_1$ and $G_2$. The first is a merged graph $\hat{G}_{1,2}$ obtained by integrating $G_1$ and $G_2$ into a single graph. The other two are graphs $\hat{G}_1$ and $\hat{G}_2$, which are projections of $G_1$ and $G_2$ onto $\hat{G}_{1,2}$. Specifically, the merged graph $\hat{G}_{1,2}$ of $G_1$ and $G_2$ is obtained as follows:

##### Definition 4

(Merged graph) Given two graphs $G_{1}(V_1, E_1)$ and $G_{2}(V_2, E_2)$, the merged graph of $G_1$ and $G_2$ is defined as $\hat{G}_{1, 2}(\hat{V}, \hat{E})$, where $\hat{V} = V_1 \cup V_2$ and $\hat{E} = E_1 \cup E_2$. In the merged graph $\hat{G}$, $v_i \in V_1$ has an updated degree $\hat{d}_i$, which is defined as

3 $$\begin{aligned} \hat{d}_i = {\left\{ \begin{array}{ll} \frac{d_i + d_j}{2} & (\exists v_j \in V_2 \textit{ s.t. } v_i \leftrightarrow v_j), \\ d_i & (\text {otherwise}). \end{array}\right. } \end{aligned}$$

In the merged graph $\hat{G}$, the degree of $v_j \in V_2$, denoted by $\hat{d}_j$, is also updated as

4 $$\begin{aligned} \hat{d}_j = {\left\{ \begin{array}{ll} \frac{d_j + d_i}{2} & (\exists v_i \in V_1 \textit{ s.t. } v_j \leftrightarrow v_i), \\ d_j & (\text {otherwise}). \end{array}\right. } \end{aligned}$$

Note that the degrees given by Equations (3) and (4) are theoretical values used to compute the QJS divergence. Thus, they do not necessarily correspond to the number of edges present in the merged graph.

From Definition 4, the projected graphs $\hat{G}_{1}$ and $\hat{G}_{2}$ are derived as $\hat{G}_{1}(\hat{V}, E_1 \cap \hat{E})$ and $\hat{G}_{2}(\hat{V}, E_2 \cap \hat{E})$, respectively. In the projected graph $\hat{G}_{1}$, the nodes from $G_{1}$ have the same degree as they had in $G_{1}$, and the same is also true for $\hat{G}_{2}$ and $G_{2}$.

**(Step 3) Computing the QJS divergence between **$G_1$** and** $G_2$: In this step, GESim computes the QJS divergence in the Similarity Calculator module, and it outputs a similarity between $G_1$ and $G_2$. By using the merged and projected graphs obtained in Step 2, the QJS divergence is derived as follows:

##### Definition 5

(Quantum Jensen-Shannon (QJS) divergence) Given graphs $G_1$ and $G_2$, their QJS divergence $D_{QJS}(\hat{G}_{1,2}, \hat{G}_1, \hat{G}_2)$ is computed by

5 $$\begin{aligned} D_{QJS}(\hat{G}_{1,2}, \hat{G}_{1}, \hat{G}_{2}) = \mathcal {H}_{1}(\hat{G}_{1,2}) - \frac{\mathcal {H}_{1}(\hat{G}_{1}) + \mathcal {H}_{1}(\hat{G}_{2})}{2}. \end{aligned}$$

QJS divergence takes a value between 0 and 1. Definition 5 indicates that QJS divergence measures how much the entropy increases by merging the two graphs $G_1$ and $G_2$ into a single graph $\hat{G}_{1,2}$. If $G_1$ and $G_2$ are isomorphic, $\hat{G}_{1,2}$ is also isomorphic to $G_1$ and $G_2$ from Definition 4, meaning that their QJS divergence is 0. In contrast, their QJS divergence is 1 if $G_1$ and $G_2$ are completely different, *i.e.,* if the graphs have no common subgraphs.

Finally, as shown in Fig. 1 (a), GESim outputs the similarity between $G_1$ and $G_2$ on the basis of the QJS divergence. Specifically, the similarity is computed by subtracting the QJS divergence from 1. That is, GESim outputs a similarity close to 1 for similar compounds.

### Evaluation on two benchmarks for similarity measures

To evaluate the characteristics of GESim as a molecular similarity measure, two benchmark datasets were used: one based on structural similarity and the other on functional similarity.[16, 17, 41] Five fingerprints were evaluated for comparison: ECFP[12], FCFP[12], APFP[10], TTFP[11], and MACCS[9]. A diameter of four and a fixed length of 2048 bits were applied for ECFP and FCFP. The Tanimoto coefficient[8] was used to measure the molecular similarity of the five fingerprints. All fingerprints were calculated via RDKit 2023.9.1[44]. The Python scripts needed to reproduce the benchmark results are available at https://github.com/ycu-iil/gesim_experiment.

#### Structural similarity benchmark

The structural similarity benchmark consists of single-assay and multi-assay datasets, which test the ability of similarity measures to rank very close analogs and diverse molecular structures, respectively[17]. The two datasets were created on the basis of the assumption that molecules with similar properties are structurally similar, which is related to the similar property principle[46]. In the datasets, a property refers to a biological activity against a target protein. The single-assay and multi-assay datasets contained 1000 repetitions of 4563 and 3629 series, respectively. A series consists of five molecules, with the most active one set as the reference and the others arranged in descending order of activity. Using the ChEMBL 20 database[5], a series of single-assay and multi-assay datasets were extracted from one and four medicinal chemistry papers, respectively. The Spearman’s rank correlation coefficient was used to compare the ranking performances of the six similarity measures. Detailed descriptions of the method of preparing the benchmark can be found in the original paper[17].

#### Functional similarity benchmark

The benchmark for ligand-based virtual screening[16, 41] consists of 118 target lists of actives and decoys from three databases: 21 targets from the directory of useful decoys (DUD)[47], 17 from the maximum unbiased validation (MUV)[48], and 80 from ChEMBL[49]. The target lists of DUD, MUV, and ChEMBL contained 31–365 actives and 1,344–15,560 decoys; 30 actives and 15,000 decoys; and 100 actives and 10,000 decoys, respectively. Virtual screening experiments were performed with 50 repetitions, each using five randomly sampled query actives. In the experiments, the remaining actives and decoys were ranked by their maximum similarity to the query actives, a method known as MAX fusion[50]. In this study, performance was evaluated via Boltzmann-enhanced discrimination of the receiver operating characteristic (BEDROC), the enrichment factor (EF), and the area under the curve (AUC), which are recommended methods for evaluating virtual screening performance[16]. Following the previous study, BEDROC at $\alpha =20$ and 100,  and EF at 1% and 5% were used. Detailed descriptions for preparing the benchmark can be found in the original paper[16].

### Calculation time comparison

To demonstrate that GESim can compute molecular similarity on a practical timescale, its computation speed was compared with those of ECFP, a representative fingerprint-based method, and GED, a graph-based method. The implementation of the vanilla GED was based on a script provided by Jensen on GitHub Gist[51], which utilizes RDKit and NetworkX. A dataset of 1,000 molecules was obtained from the ZINC database to measure the computation time. The first molecule was used as the reference molecule, and the computation time for similarity calculations against the 1,000 molecules, including the reference molecule, was measured. The measurement was repeated ten times; the timeout for a single similarity calculation was set to 0.1 s as a threshold for the practical computation time; and the mean and standard deviation were calculated to compare the three methods. Since the GED implementation does not support bulk similarity calculations, RDKit and GESim were evaluated under the same conditions by computing the similarity values for each molecule individually, without using their bulk calculation functionalities, to ensure a fair assessment. As for the ECFP evaluation, similarity calculations using pre-computed ECFP fingerprints were also performed in the experiment. A Python script for reproducing the comparison is available at https://github.com/ycu-iil/gesim_experiment.

## Results and discussion

### Evaluation on structural similarity benchmark

**Fig. 2:**
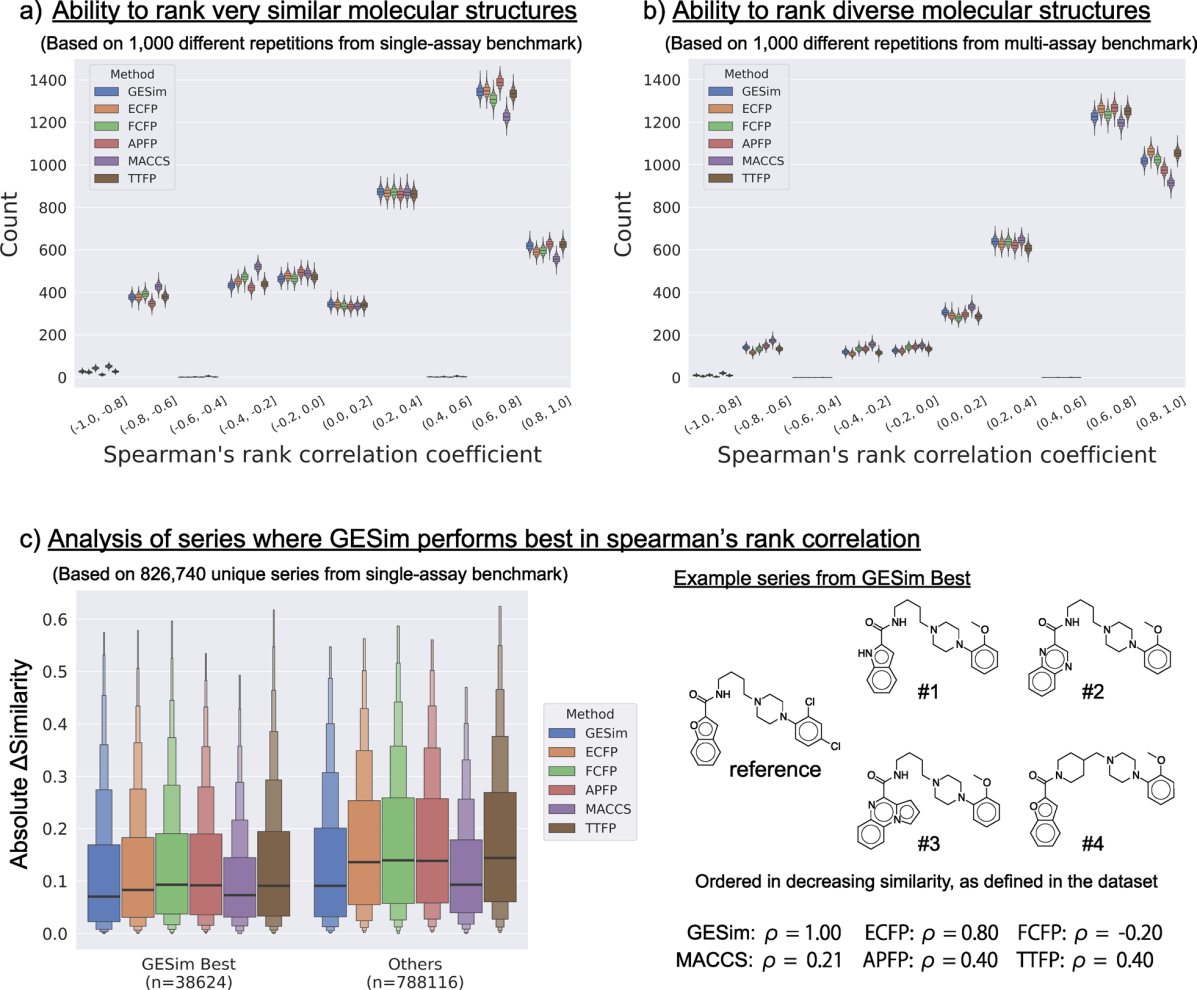
Performance of six molecular similarity measures on two structural similarity benchmarks: (**a**) single-assay and (**b**) multi-assay benchmarks, each with 1,000 different repetitions. The Spearman’s rank correlation coefficient ($\rho$) was calculated to assess the ability to reproduce the benchmark series orders. The correlation coefficients were grouped into bins with a width of 0.2, and the distributions within each bin were visualized using a boxen plot to facilitate the comparison of performance across the measures. **c** A boxen plot visualizes the comparison between GESim best and others in terms of molecular similarity within each series (absolute $\Delta$similarity). GESim best refers to the 38,624 series (out of 826,740 unique series in the single-assay benchmark) where GESim achieved the highest Spearman’s rank correlation coefficient, while others include the remaining series. Absolute $\Delta$similarity represents the similarity within a series, calculated as the absolute difference between the similarity of the reference molecule to the first and last molecules in a series. To facilitate comparison, 5% of the data are excluded as outliers in the boxen plot (c). An example series from GESim best is shown with the corresponding Spearman’s rank correlation coefficients ($\rho$) for each method displayed underneath (lower right panel). GESim, ECFP, FCFP, APFP, MACCS, and TTFP are shown in blue, orange, green, red, purple, and brown, respectively

To assess the ability of GESim to order molecules by structural similarity, two benchmark datasets, single-assay and multi-assay benchmarks, were used[17]. Figure 2a and 2b shows the performance of GESim and five representative molecular similarity measures in reproducing the benchmark series orders for single-assay (4,563 series) and multi-assay (3,629 series) benchmarks in 1,000 different repetitions. The Spearman’s rank correlation coefficients from each repetition were grouped into bins with a width of 0.2, and the distributions within each bin were visualized using a boxen plot to facilitate the comparison of performance across the six measures.

As reported in a previous study[17], APFP showed the best performance on the single-assay benchmark and reproduced or almost reproduced an average of 626 original series orders with a coefficient of 0.8 or higher in this evaluation. GESim demonstrated comparable performance to that of APFP, reproducing or almost reproducing an average of 618 original series orders. On the other hand, ECFP achieved the best performance on the multi-assay benchmark and reproduced or almost reproduced an average of 1,061 original series orders with a coefficient of 0.8 or higher, as reported in a previous study[17]. GESim demonstrated intermediate performance among the six measures, reproducing and almost reproducing an average of 1,018 original series orders. Its performance surpassed that of APFP, which obtained 974 original series orders. These results suggest that GESim has intermediate characteristics between those of APFP and ECFP. Additionally, we compared the two groups, series where GESim performed best (GESim Best) and those where it did not (Others), in terms of the similarity between molecules within each series, as shown in Fig. 2c (left panel). To quantify this, absolute $\Delta$similarity was defined as a similarity within a series by calculating the absolute difference between the similarity of the reference molecule to the first and last molecules in a series. The result indicates that series where GESim exhibited the highest ranking performance tend to have lower absolute $\Delta$similarity compared to the other series. This suggests that GESim has the ability to differentiate between highly similar molecules, even in cases where other methods fail to effectively distinguish their similarity. To illustrate the characteristics of GESim best series, an example series from GESim best is shown in Fig. 2c (right panel), with the corresponding Spearman’s rank correlation coefficients ($\rho$) for each method displayed underneath. Complete information on all series included in GESim best is provided as a CSV file in the Supplementary file.

**Fig. 3:**
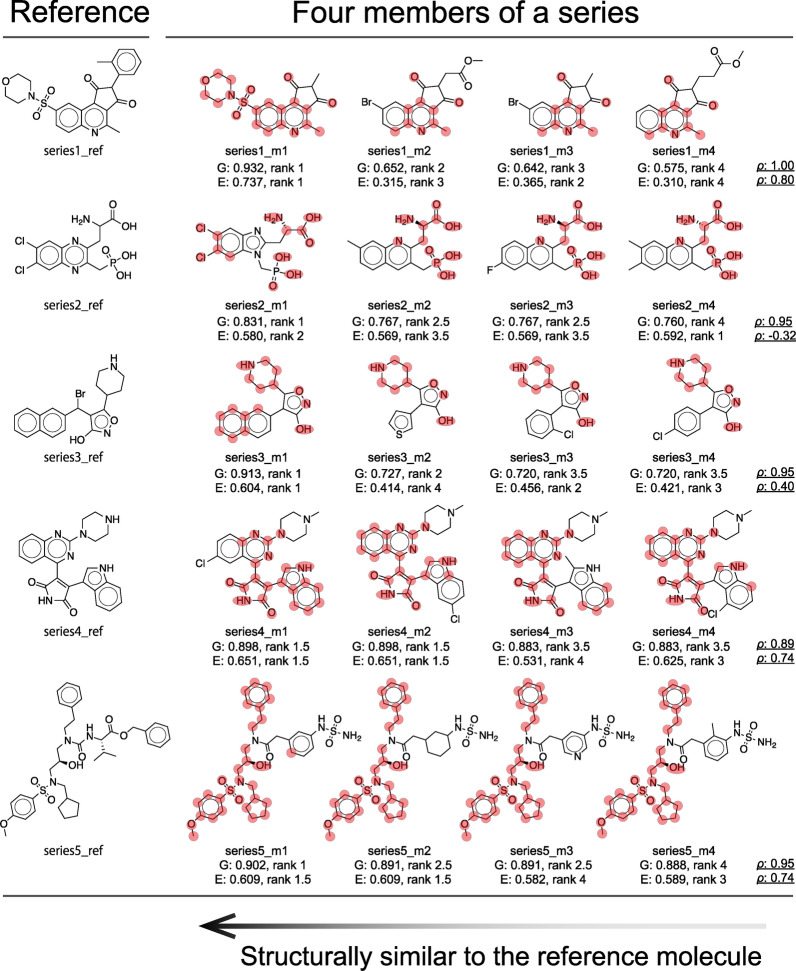
Visualization of atom-pair matching performed by the Graph Aligner module. The red-highlighted atoms within each molecule represent those that match atoms in the reference molecule. Five molecules in a series from the structural similarity benchmark are positioned horizontally, where the first molecule serves as the reference and the next four are ordered on the basis of their similarity to this reference. The values labeled “G” and “E” below each molecule denote the similarity scores calculated by GESim and the Tanimoto similarity using ECFP, respectively, in relation to the reference molecule. The rankings in descending order based on these scores are also indicated, along with Spearman’s rank correlation coefficient ($\rho$) computed from these rankings

To visually confirm how GESim identifies atom-pair matches and nonmatches between the reference molecule and those of a series of four molecules during the similarity calculation, subgraph matching visualizations (described for the Graph Aligner module in Fig. 1) were performed and are shown in Fig. 3.

In the top series in Fig. 3, the second to fourth molecules have the same atom-pair matching with the reference molecule, but their similarity values with respect to the reference molecule differ, successfully reproducing the order of the original series. This ability to reflect such subtle differences in similarity values can be attributed to the vNGE algorithm, which considers the degrees of the atoms in a molecule. As previously noted, vNGE sensitively captures differences in the inherent structural complexity of graphs, especially the degree distributions of nodes; if a molecule has a degree distribution close to that of the reference molecule, GESim tends to consider it more similar than other molecules. This is why GESim can distinguish two molecules even if they have the same atom-pair matching. For example, in series 1 of Fig. 3, molecules m2, m3, and m4 share an identical matched subgraph. However, GESim considers differences in the degree distributions of these molecules, leading to distinct similarity scores. In contrast, when both the matched subgraph and degree distribution are identical, the similarity scores are identical, as observed for m3 and m4 in series 3 of Fig. 3.

Additionally, the visualizations provide insights into potential improvements to the GESim algorithm, such as subgraph matching. By examining the matched atom pairs in the five series, some cases can be observed in which atoms that are intuitively expected to match are instead identified as nonmatches. This may be because the Graph Aligner module uses atom-fingerprint-based subgraph matching. However, we believe that this problem can be solved by using other methods or by combining several methods to achieve subgraph matching that is closer to expert-level intuition. Figure S3 illustrates that while MCS-based subgraph matching (as implemented in RDKit with default settings) provides more intuitive matching results, GESim utilizing our subgraph matching approach achieves better ranking performance across the five evaluated series. Additionally, GESim with MCS-based subgraph matching significantly increases computational time, making it up to 58,000 times slower than the current GESim implementation, as shown in Supplementary Fig. S4. Given its computational efficiency, our subgraph matching approach could be applied to tasks such as similarity search, hierarchical clustering, and molecule alignment, where MCS is commonly used but not always required for identifying the maximum common substructure. Considering user convenience, integrating MCS algorithms implemented in RDKit into GESim could provide greater flexibility in adjusting subgraph matching conditions for specific use cases, potentially improving alignment with chemical intuition and making it a valuable direction for future development. In addition, it is possible to improve the quality of subgraph matching by using node embedding methods such as CONE [52], albeit at the cost of some additional computation time.

### Evaluation on Functional Similarity Benchmark

**Fig. 4:**
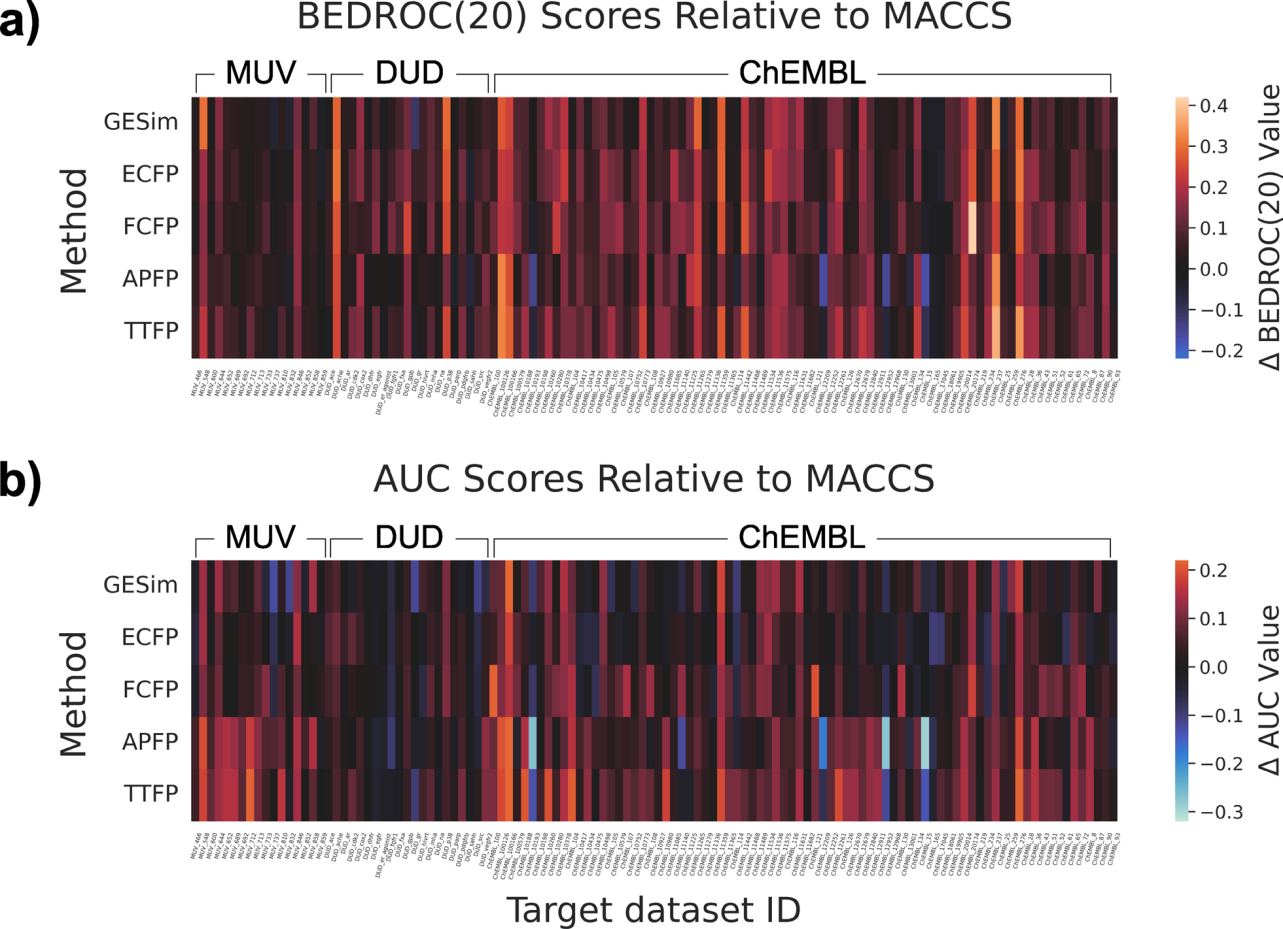
Average performance of six molecular similarity measures with (**a**) BEDROC($\alpha =20$) and (**b**) AUC on the ligand-based virtual screening benchmark. The performance differences ($\Delta$BEDROC(20) and $\Delta$AUC values) between MACCS and each of the five similarity measures (GESim, ECFP, FCFP, APFP, and TTFP) were visualized using a heatmap. Red indicates better performance compared with the MACCS, while blue indicates worse performance. The plot of BEDROC($\alpha =100$) is provided in Fig. S1. The raw values used in the plots are available as CSV files in the Supporting Information

The average performance of GESim on the ligand-based virtual screening benchmark, which focuses on functional similarity, created by Landrum and Riniker[16] was tested in comparison with those of the five molecular similarity measures, as shown in Fig. 4.

**Fig. 5:**
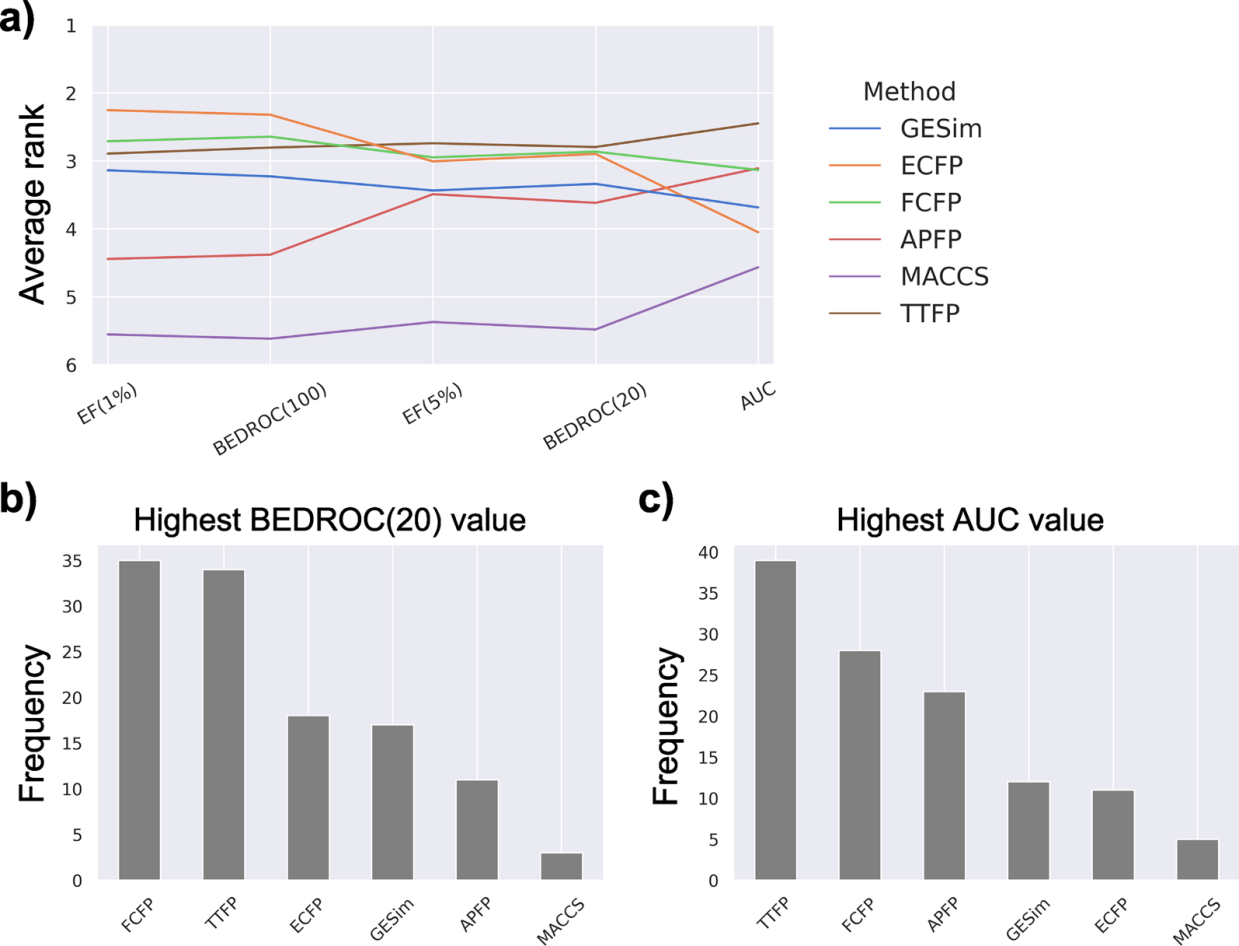
Statistical analysis of the ligand-based virtual screening benchmark. **a** Average rank performance of six molecular similarity measures across 118 targets. GESim, ECFP, FCFP, APFP, MACCS, and TTFP are shown in blue, orange, green, red, purple, and brown, respectively.** b** The highest BEDROC($\alpha =20$) count and** c** the highest AUC count across 118 targets are shown as bar plots. The bar plots of BEDROC($\alpha =20$), EF(1%), and EF(5%) are shown in Fig. S2

To facilitate the comparison, the performance differences between each of the five similarity measures (GESim, ECFP, FCFP, APFP, and TTFP) and MACCS, which was used as a baseline in the previous study[16], were visualized using a heatmap. A visual inspection shows that the five methods displayed roughly similar performance, each showing certain advantages depending on the target, and GESim outperformed the other methods for certain targets (MUV 466, ChEMBL 10475, and ChEMBL 11265). With respect to AUC performance, GESim did not demonstrate outstanding performance for any particular target and yielded average results overall. The plot of BEDROC($\alpha =100$) is provided in Fig. S1; the plots of EF(1%) and EF(5%) are not depicted because the maximum EF values vary for each target, and comparisons between targets are difficult. As observed in the heatmaps, GESim generally exhibits lower performance on the MUV dataset compared to the other methods, as indicated by the prevalence of black and blue regions. This is consistent with the fact that the MUV dataset was constructed by removing trivial structural analogues from the dataset, which naturally disadvantages graph-based similarity measures like GESim. To further examine these results, we analyzed the average rank performance for each evaluation metric and the highest performance count across 118 targets for each metric, as illustrated in Fig. 5.

Consistent with the previous study, FCFP and TTFP performed well, whereas MACCS had the lowest performance. For both the average rank performance and the number of top-performing results, GESim consistently ranked in the middle for all the evaluation metrics, indicating that GESim did not exhibit outstanding performance on this virtual screening benchmark. The bar plots of BEDROC($\alpha =20$), EF(1%), and EF(5%) are shown in Fig. S2. Regarding ECFP and APFP, ECFP outperformed its counterparts according to BEDROC(20), whereas APFP outperformed the other methods in terms of AUC. Since GESim’s performance is between those of these two methods, as is also indicated by the structural similarity benchmark, these findings imply that GESim may possess characteristics intermediate between those of APFP and ECFP.

### Calculation time comparison

**Fig. 6:**
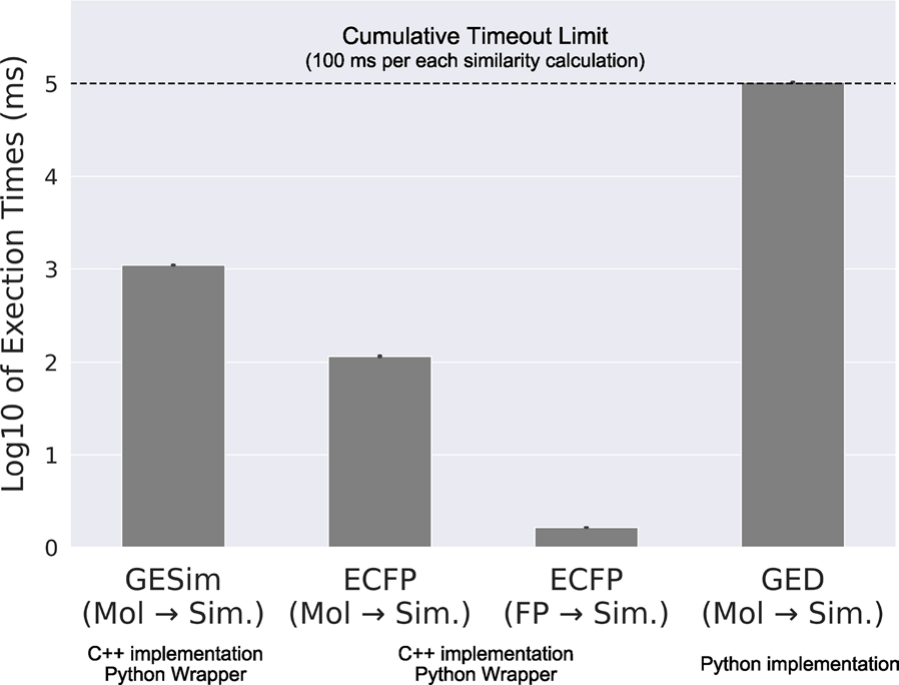
Calculation time comparison between GESim, ECFP with Tanimoto similarity, and GED. As for ECFP, the times of similarity computations using on-the-fly computed and pre-computed ECFP are included. The bar plots show the mean computation time with the standard deviation for each method across 10 trials. The vertical axis represents the logarithm of the total computation time in milliseconds. The dotted line represents the logarithm of the timeout limit

The average computation times for calculating 1,000 molecular similarities via three methods—GESim, ECFP, and GED—are shown in Fig. 6. GESim computes the 1,000 molecule similarities in a mean time of 1.098 s, which is approximately 10 times slower than the similarity computation using on-the-fly computed ECFP (0.116 s) and about 550 times slower than using pre-computed ECFP (0.002 s). Conversely, GED required 0.1 s—the threshold time set for each calculation—for almost all similarity calculations; thus, there is no guarantee that the optimal GED values were obtained.

These results, along with the fact that GESim completed the two benchmark computations as well as did the other methods, indicate that GESim can be used in practical cases as a graph-based molecular similarity calculation method. Note that the observed computation times may be significantly affected by the processing speeds of the programming languages used rather than by the inherent differences in the algorithms themselves. The algorithms of GESim and ECFP were implemented in C++, whereas that of GED was implemented in Python. Although GED with extended reduced graphs as a molecular representation, as reported in a previous study[18], would be appropriate for practical application, this implementation is not publicly available.

## Conclusion

In this study, we introduced GESim, an ultrafast graph-based method for calculating molecular similarity, and demonstrated its applicability in structural and functional similarity assessments, where fingerprint-based methods have traditionally been employed. By using vNGE, GESim enables graph-based molecular similarity calculations at a computational speed comparable to those of fingerprint-based methods and considers entire molecular graphs. From the two benchmark results, GESim appears to have characteristics intermediate between those of APFP and ECFP and to have the ability to differentiate between highly similar molecules, even in cases where other methods fail to effectively distinguish their similarity. On the basis of these findings, GESim may pave the way for graph-based similarity calculation methods in tasks, such as virtual screenings and database searches.

## Supplementary Information

Supplementary file 1.

## Data Availability

The GESim package is publicly available on GitHub at https://github.com/LazyShion/GESim under the MIT License. The README file in the GitHub repository provides information about how to install and use the package. The Python scripts needed to reproduce the benchmark results are available at https://github.com/ycu-iil/gesim_experiment.
