# Supplementary material for: GESim: ultrafast graph-based molecular similarity calculation via von Neumann graph entropy
Source: J Cheminform. 2025 Apr 22;17:57. doi: 10.1186/s13321-025-01003-6 (PMC12013028; doi:10.1186/s13321-025-01003-6)
Supplement: Supplementary file 1 [file 13321_2025_1003_MOESM1_ESM.pdf]

Supporting Information for GESim: Ultrafast Graph-Based Molecular Similarity Calculation via von Neumann Graph Entropy

Hiroaki Shiokawa,[*,†,‡,§] Shoichi Ishida,[*,¶,‡,§] and Kei Terayama[*,¶,‡]

†*Center for Computational Sciences, University of Tsukuba, Tennodai 1-1-1, Tsukuba, Ibaraki, 305-8577, Japan*

‡*MolNavi LLC, #402 Wizard building 1-4-3 Sengen-cho Nishi-ku, Yokohama 220-0072 Kanagawa, Japan*

¶*Graduate School of Medical Life Science, Yokohama City University, 1-7-29, Suehiro-cho, Tsurumi-ku, Yokohama 230-0045 Kanagawa, Japan*

§*These authors contributed equally to this work*

E-mail: shiokawa@cs.tsukuba.ac.jp; ishida.sho.nm@yokohama-cu.ac.jp; terayama@yokohama-cu.ac.jp

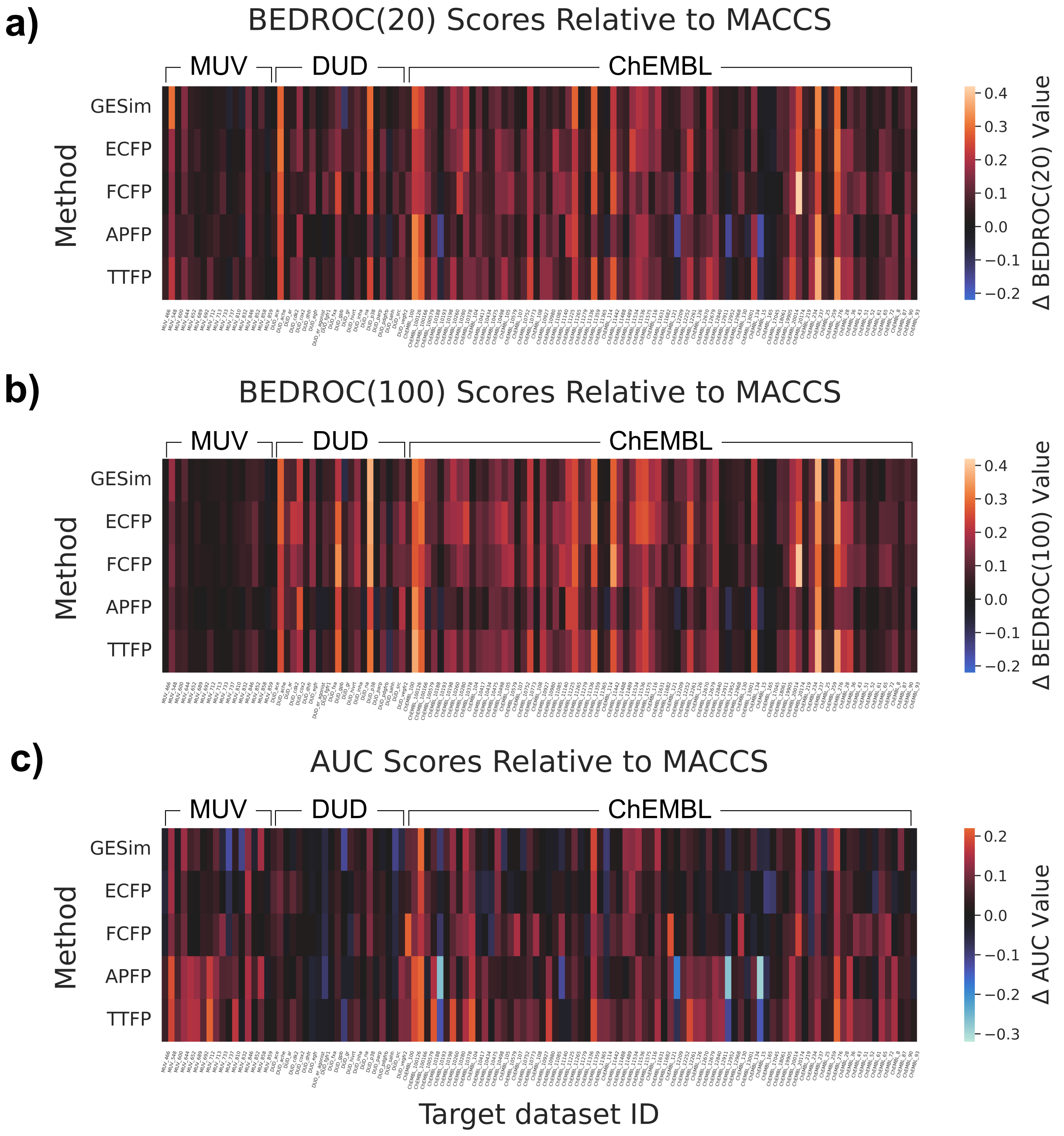

Figure S1: Average performance of six molecular similarity measures with (a) BEDROC($\alpha = 20$), (b) BEDROC($\alpha = 100$), and (c) AUC on the ligand-based virtual screening benchmark. The performance differences between each of the five similarity measures (GESim, ECFP, FCFP, APFP, and TTFP) and MACCS were visualized using a heatmap. Red indicates better performance compared with the MACCS, while blue indicates worse performance. The raw values used in the plots are available as CSV files in the Supporting Information.

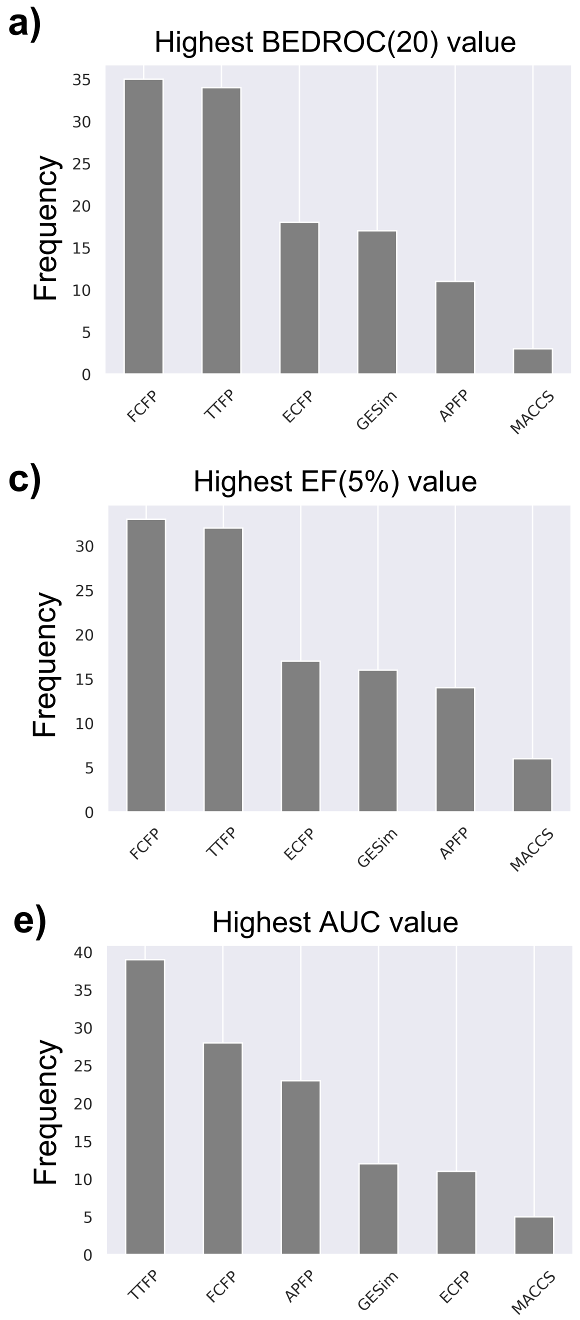

Figure S2: Statistical analysis of the ligand-based virtual screening benchmark. (a) The highest BEDROC($\alpha = 20$) count, (b) highest BEDROC($\alpha = 100$) count, (c) highest EF(5%) count, (d) highest EF(1%) count, and (e) highest AUC count across 118 targets are shown as bar plots.

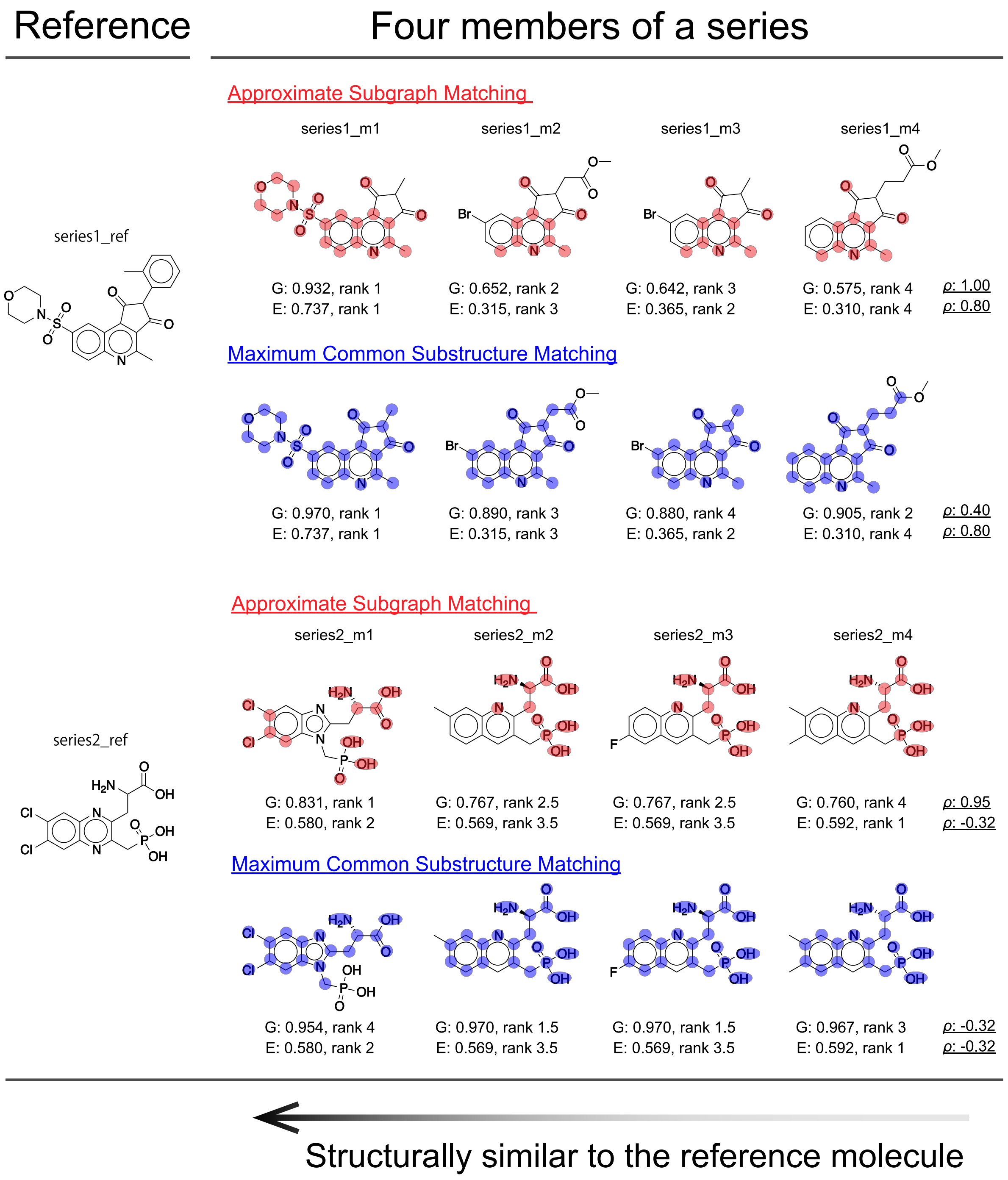
Reference
Four members of a series
Approximate Subgraph Matching
series1_m1
series1_m2
series1_m3
series1_m4
series1_ref
G: 0.932, rank 1
E: 0.737, rank 1
G: 0.652, rank 2
E: 0.315, rank 3
G: 0.642, rank 3
E: 0.365, rank 2
G: 0.575, rank 4
E: 0.310, rank 4
ρ: 1.00
ρ: 0.80
Maximum Common Substructure Matching
G: 0.970, rank 1
E: 0.737, rank 1
G: 0.890, rank 3
E: 0.315, rank 3
G: 0.880, rank 4
E: 0.365, rank 2
G: 0.905, rank 2
E: 0.310, rank 4
ρ: 0.40
ρ: 0.80
Approximate Subgraph Matching
series2_m1
series2_m2
series2_m3
series2_m4
series2_ref
G: 0.831, rank 1
E: 0.580, rank 2
G: 0.767, rank 2.5
E: 0.569, rank 3.5
G: 0.767, rank 2.5
E: 0.569, rank 3.5
G: 0.760, rank 4
E: 0.592, rank 1
ρ: 0.95
ρ: -0.32
Maximum Common Substructure Matching
G: 0.954, rank 4
E: 0.580, rank 2
G: 0.970, rank 1.5
E: 0.569, rank 3.5
G: 0.970, rank 1.5
E: 0.569, rank 3.5
G: 0.967, rank 3
E: 0.592, rank 1
ρ: -0.32
ρ: -0.32
Structurally similar to the reference molecule

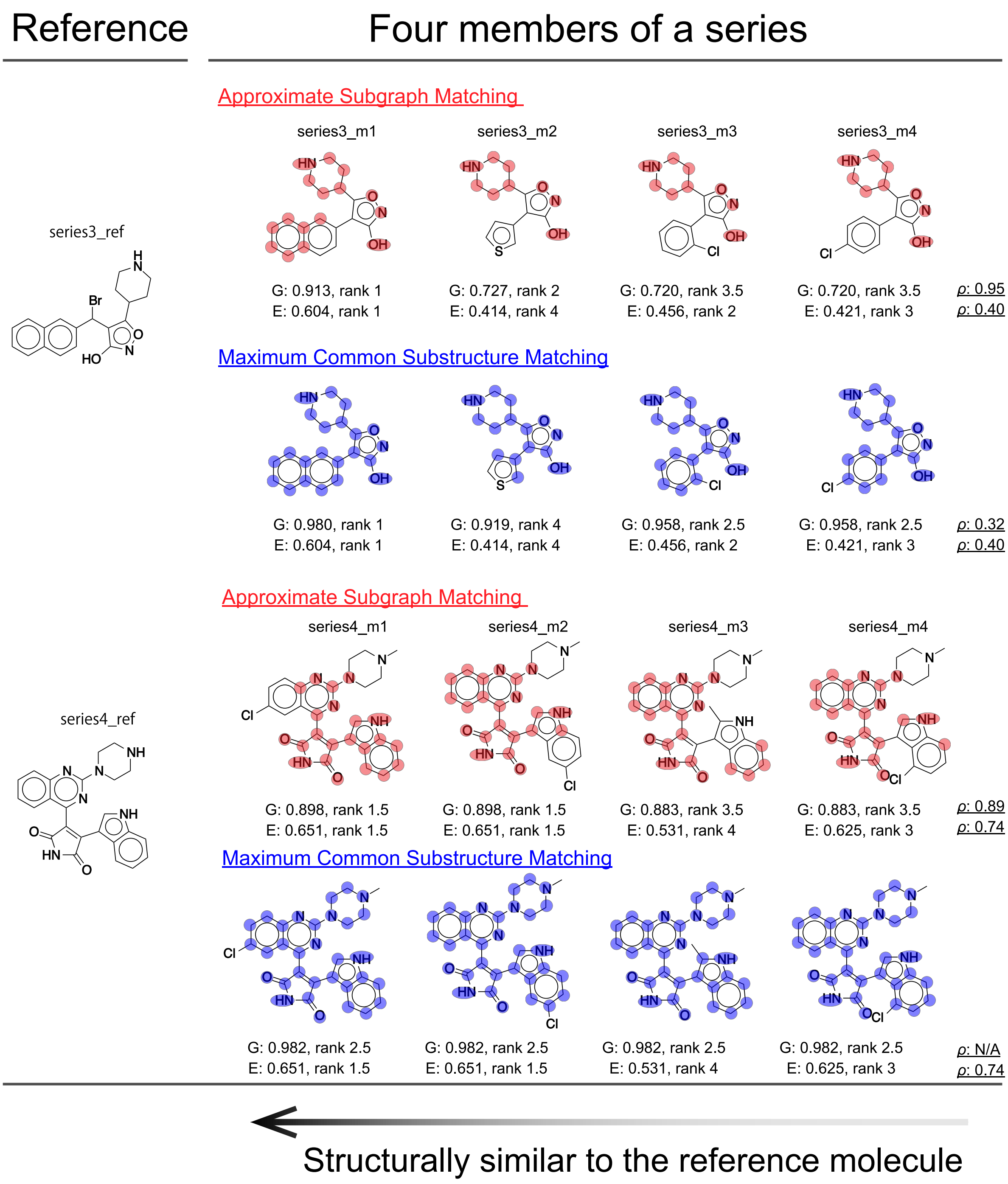
Reference
Four members of a series
Approximate Subgraph Matching
series3_m1
series3_m2
series3_m3
series3_m4
series3_ref
G: 0.913, rank 1
E: 0.604, rank 1
G: 0.727, rank 2
E: 0.414, rank 4
G: 0.720, rank 3.5
E: 0.456, rank 2
G: 0.720, rank 3.5
E: 0.421, rank 3
ρ: 0.95
ρ: 0.40
Maximum Common Substructure Matching
G: 0.980, rank 1
E: 0.604, rank 1
G: 0.919, rank 4
E: 0.414, rank 4
G: 0.958, rank 2.5
E: 0.456, rank 2
G: 0.958, rank 2.5
E: 0.421, rank 3
ρ: 0.32
ρ: 0.40
Approximate Subgraph Matching
series4_m1
series4_m2
series4_m3
series4_m4
series4_ref
G: 0.898, rank 1.5
E: 0.651, rank 1.5
G: 0.898, rank 1.5
E: 0.651, rank 1.5
G: 0.883, rank 3.5
E: 0.531, rank 4
G: 0.883, rank 3.5
E: 0.625, rank 3
ρ: 0.89
ρ: 0.74
Maximum Common Substructure Matching
G: 0.982, rank 2.5
E: 0.651, rank 1.5
G: 0.982, rank 2.5
E: 0.651, rank 1.5
G: 0.982, rank 2.5
E: 0.531, rank 4
G: 0.982, rank 2.5
E: 0.625, rank 3
ρ: N/A
ρ: 0.74
Structurally similar to the reference molecule

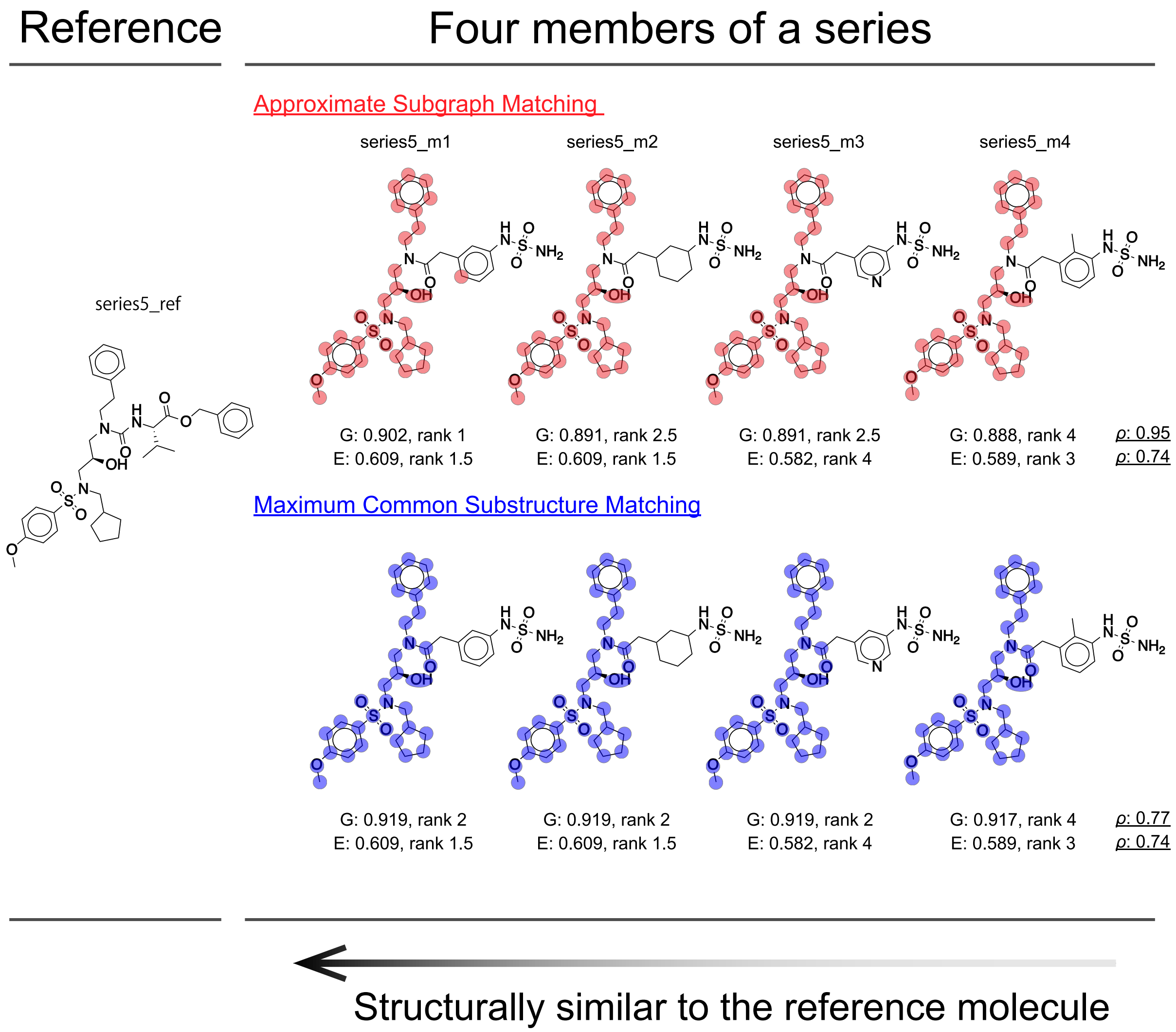

Figure S3: (pages ranging from 4 to 6) Comparison of atom-pair matching between GESim and its variant with MCS instead of GraphAligner (hereafter called MCS). The red- and blue-highlighted atoms within each molecule represent those that match atoms in the reference molecule using GraphAligner and MCS, respectively. Five molecules in a series from the structural similarity benchmark are positioned horizontally, where the first molecule serves as the reference and the next four are ordered on the basis of their similarity to this reference. The values labeled "G" and "E" below each molecule denote the similarity scores calculated by GESim and the Tanimoto similarity using ECFP, respectively, in relation to the reference molecule. The rankings in descending order based on these scores are also indicated, along with Spearman's rank correlation coefficient (ρ) computed from these rankings.

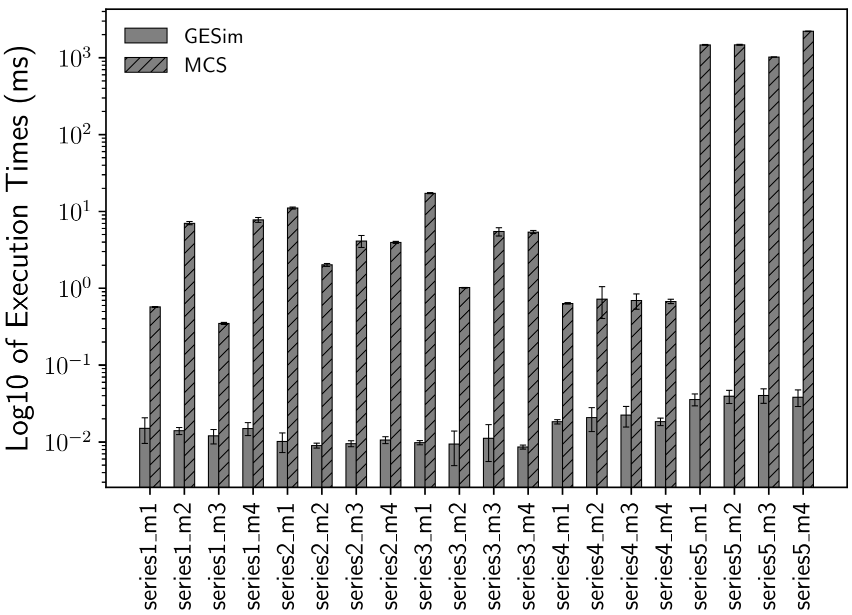

Figure S4: Calculation time comparison between GESim and its variant with MCS instead of GraphAligner (hereafter called MCS). The bar plots show the mean computation time with the standard deviation for each method across 10 trials. The vertical axis represents the logarithm of the total computation time in milliseconds.
